# Nine-year incident diabetes is predicted by fatty liver indices: the French D.E.S.I.R. study

**DOI:** 10.1186/1471-230X-10-56

**Published:** 2010-06-07

**Authors:** Beverley Balkau, Celine Lange, Sylviane Vol, Frederic Fumeron, Fabrice Bonnet

**Affiliations:** 1 INSERM CESP Center for Research in Epidemiology and Population Health, U1018, Epidemiology of diabetes, obesity and chronic kidney disease over the lifecourse, Villejuif, France; 2 University Paris-Sud 11, UMRS 1018, Villejuif, France; 3 IRSA, La Riche, France; 4 INSERM, U695, Genetic determinants for type 2 diabetes and its vascular complications, Xavier Bichat School of Medicine, Paris, France; 5 University Paris Diderot-Paris 7, Paris, France; 6 Service Endocrinologie, CHU Rennes, Université Rennes 1, Hôpital Sud, Rennes, France; 7 INSERM U625, Rennes, France

## Abstract

**Background:**

Fatty liver is known to be linked with insulin resistance, alcohol intake, diabetes and obesity. Biopsy and even scan-assessed fatty liver are not always feasible in clinical practice. This report evaluates the predictive ability of two recently published markers of fatty liver: the Fatty Liver Index (FLI) and the NAFLD fatty liver score (NAFLD-FLS), for 9-year incident diabetes, in the French general-population cohort: Data from an Epidemiological Study on the Insulin Resistance syndrome (D.E.S.I.R).

**Methods:**

At baseline, there were 1861 men and 1950 women, non-diabetic, aged 30 to 65 years. Over the follow-up, 203 incident diabetes cases (140 men, 63 women) were identified by diabetes-treatment or fasting plasma glucose ≥ 7.0 mmol/l. The FLI includes: BMI, waist circumference, triglycerides and gamma glutamyl transferase, and the NAFLD-FLS: the metabolic syndrome, diabetes, insulin, alanine aminotransferase, and asparate aminotransferase. Logistic regression was used to determine the odds ratios for incident diabetes associated with categories of the fatty liver indices.

**Results:**

In comparison to those with a FLI < 20, the age-adjusted odds ratio (95% confidence interval) for diabetes for a FLI ≥ 70 was 9.33 (5.05-17.25) for men and 36.72 (17.12-78.76) for women; these were attenuated to 3.43 (1.61-7.28) and 11.05 (4.09 29.81), after adjusting on baseline glucose, insulin, hypertension, alcohol intake, physical activity, smoking and family antecedents of diabetes; odds ratios increased to 4.71 (1.68-13.16) and 22.77 (6.78-76.44) in those without an excessive alcohol intake. The NAFLD-FLS also predicted incident diabetes, but with odds ratios much lower in women, similar in men.

**Conclusions:**

These fatty liver indexes are simple clinical tools for evaluating the extent of liver fat and they are predictive of incident diabetes. Physicians should screen for diabetes in patients with fatty liver.

## Background

Non-alcoholic fatty liver disease (NAFLD) is the most common cause of chronic liver injury in Western countries and about 20 to 30% of people with NAFLD have non alcoholic steatohepatitis (NASH), which is associated with fibrosis and can progress to cirrhosis, terminal liver failure and hepatocellular carcinoma [1]. The obese, the insulin resistant and the diabetic patient have a higher prevalence of NAFLD [2], and metabolic steatosis may be a major risk factor for disease progression. A liver biopsy is the only way to reliably diagnose NAFLD, but even this technique is not 100% precise due to the sampling variability of biopsies. Clearly a biopsy is very invasive and carries a risk, thus proxy markers are often used [3]. However while the extent of liver fat correlates with liver enzymes: alanine aminotransferase (ALT), asparate aminotransferase (AST), gamma glutamyl transferase (GGT), there are other commonly measured factors such as obesity which are also associated. Thus to aid in screening for fatty liver, Bedogni et al. [4], developed a simple score, from a cohort of patients with suspected liver disease and a group of controls. Their Fatty Liver Index (FLI) uses an equation with GGT, triglycerides, body mass index (BMI) and waist circumference. Another score has recently been published by a Finnish group [5,6], the NAFLD fatty liver score (NAFLD-FLS). This includes in its definition, the presence of diabetes, the metabolic syndrome, and levels of ALT, AST and fasting insulin.

Cross-sectional studies have shown that fatty liver and metabolic abnormalities, including insulin resistance and diabetes, occur together [7]. We have recently shown, in a cross-sectional study, that the FLI is positively associated with insulin resistance, coronary risk and with an increased intima-media thickness [8]. It is generally thought that insulin resistance precedes the development of fatty liver, but the evidence is not conclusive, and it has been proposed that fatty liver is pathogenetically involved in the emergence of insulin resistance and type 2 diabetes [9].

A number of prospective studies have shown that elevations in liver enzymes ALT and GGT precede the development of diabetes [10,11]. Whether NAFLD precedes diabetes, using a more precise evaluation of NAFLD, has been much less studied. In a cohort of Korean adults, fatty liver, as assessed by ultrasound, was predictive of diabetes, particularly moderate to severe fatty liver, after adjusting for other diabetes risk factors [12]. In a 4-year prospective Chinese case-control study in which NAFLD was evaluated by ultrasound, it was shown that NAFLD preceded metabolic disorders, independently of obesity [13]. To our knowledge, there is no large study which has specifically assessed whether fatty liver indices predict type 2 diabetes.

We study in a prospective context, whether the Fatty Liver Index and the NAFLD fatty liver score are able to predict incident diabetes in a large French cohort, followed over a 9-year period. We studied men and women separately to evaluate whether the relations were consistent.

## Methods

### Study population

The study population was men and women aged 30-65 years, who participated in the 9 year follow-up study, Data from an Epidemiological Study on the Insulin Resistance syndrome (D.E.S.I.R.). Participants were recruited from volunteers offered periodic health examinations free of charge by the French Social Security, in 10 health examination centres in western France [14]. All participants signed an informed consent and the protocol was approved by the ethics committee of the Kremlin Bicêtre Hospital, Kremlin Bicêtre, France.

Incident cases of diabetes were identified by treatment for diabetes or a fasting plasma glucose ≥ 7.0 mmol/l, at one of the four, three-yearly examinations, after exclusion of individuals with diabetes at baseline and those with unknown diabetic status at the 9-year examination. We study the 1861 men and 1950 women who had data available at baseline on glucose, triglycerides, BMI, GGT, waist circumference and known diabetes status at the 9-year examination. For the calculation of the NAFLD-FLS, data is available for 1848 men and 1940 women.

### Measures at inclusion

Two measures of blood pressure, using a mercury sphygmomanometer, and two measures of heart rate were taken in a supine position after participants had rested during 5 minutes; mean values were used. Weight and height were measured on lightly clad participants, and BMI calculated. The waist circumference, the smallest circumference between the lower ribs and the iliac crests, was also measured.

The examining physician recorded treatment for diabetes, hypertension and lipids and whether participants had diabetes in their family; for women, they also recorded whether they had a baby over 4 kg and their menopausal status. Hypertension was defined by systolic/diastolic blood pressures of at least 140/90 mmHg or being on antihypertensive medication. Smoking habits (current smoker or not), alcohol consumption (gram per day of alcohol from wine, beer, cider, spirits) and degree of physical activity (at home, at work and sport, each one coded on a four level scale) were assessed using a self-administered questionnaire. Intense physical activity was coded if an individual had a score of four on any of the three items (at home, at work or sport), or a score of 3 for sporting activity and 3 for physical activity either at home or at work.

All biochemical measurements were from one of four health-centre laboratories located in France at Blois, Chartres, La Riche or Orléans. Fasting plasma glucose, measured by the glucose-oxidase method, was applied to fluoro-oxalated plasma using a Technicon RA100 (Bayer Diagnostics, Puteaux, France) or a Specific or a Delta device (Konelab, Evry, France). Total cholesterol, HDL-cholesterol, triglycerides, ALT, AST, GGT, and creatinine were assayed by DAX 24 (Bayer Diagnostics, Puteaux, France) or KONE (Evry, France). Insulin was quantified by micro particle enzyme immunoassay with an automated analyzer IMX (Abbott, Rungis, France). White cell counts were determined by a Technicon H* or a Technicon H3RTX (Bayer Diagnostics, Puteaux, France) or a JT2 (Beckman/Coulter, Roissy, France) or an Argos (ABX, Montpellier, France). The inter-laboratory variability was assessed monthly on normal and pathological values for each biologic variable.

### Surrogate Measures of Fatty Liver

The FLI score was developed by logistic regression using data from a population of individuals, 216 with and 280 without suspected liver disease [4]. The presence of fatty liver was identified using ultrasound, and defined according to a brightness scale. The score runs from 0 and 100 and estimates the percentage chance of having a fatty liver:

with triglycerides measured in mg/dl, GGT in IU/l and waist circumference in cm. All four factors contributed about equally to this score when converted to Z-scores.

The more recently published NAFLD Fatty Liver Score [5,6], was developed from a Finish population which included 359 non diabetic and 111 type 2 diabetic individuals; liver fat content was measured by proton magnetic resonance spectroscopy and NAFLD was defined to be present when there was at least 55.6 mg triglyceride per g. of liver tissue. The NAFLD-FLS equation is the linear function derived from logistic regression, and so could be converted to a percent chance of fatty liver, in the same form as the equation above. We retain the form of the equation as published:

with insulin measured in mU/l and AST and ALT in U/L. The International Diabetes Federation (IDF) definition of the metabolic syndrome is [15]: central obesity (waist circumference ≥ 94 cm in men, ≥ 80 cm in women) and at least two of the following factors: (1) triglycerides ≥ 1.70 mmol/l or specific treatment for this lipid abnormality; (2) HDL-cholesterol < 1.03 mmol/l in men and < 1.29 mmol/l in women or specific treatment for this lipid abnormality; (3) systolic blood pressure ≥ 130 mm Hg or diastolic blood pressure ≥ 85 mm Hg or treatment for previously diagnosed hypertension; (4) fasting plasma glucose ≥ 5.6 mmol/L or previously diagnosed type 2 diabetes. As we did not know whether lipid treatment was specific for HDL-cholesterol or for triglycerides, we did not include this in our definition. In our population, as we studied incident diabetes, there were no individuals with diabetes at baseline, so the term in type 2 diabetes does not contribute to the NAFLD-FLS in our study.

### Statistical methods

Analyses used SAS Version 9.1 (SAS Institute Inc. Cary, NC USA). BMI, fasting glucose, insulin, ALT, AST, GGT, triglycerides and white blood cell count were log-transformed for statistical analysis, because of their skewed distributions. Hypertension (HTA) was defined by a systolic blood pressure ≥ 140 mmHg and/or a diastolic blood pressure ≥ 90 mmHg and/or an antihypertensive treatment.

The characteristics of the men and women, according to FLI classes, are shown as means ± SD or numbers (percentages), which we compared using ANOVA or logistic regression, after adjusting for age. Spearman correlation coefficients were used to describe associations.

Logistic regression models were used to predict 9-year incident diabetes from three categories of the FLI, with adjustment for diabetes risk factors: age, glucose, insulin, physical activity, smoking, alcohol, diabetes in the family, and HTA. A trend test was used to compare the odds ratios across the three FLI categories, with the categories were coded as 1, 2 and 3. A secondary analysis compared the odds ratios in those without an excessive alcohol intake: < 30 g/day in men and < 20 g/day in women. To compare the two fatty liver indices, odds ratios for incident diabetes were also compared across quartiles of the indices, with and without adjustment for the above diabetes risk factors. The difference in odds ratios between men and women was tested by the inclusion of an interaction term between sex and each index. The data was further grouped to compare individuals in the upper quartile group and the three lower quartile groups, to decrease the width of the confidence intervals of the odds ratios, as there were few incident diabetes cases in the lowest quartile group. The c-statistic and the model goodness-of-fit Hosmer-Lemeshow tests were evaluated for the two indices, and compared with the predictive model for incident diabetes that we have already published [16]. The D.E.S.I.R. diabetes risk score includes, for men: waist circumference, smoking, fasting glucose and GGT, and for women: BMI, diabetes in the family, fasting glucose and triglycerides.

## Results

### Characteristics of the study population according to the Fatty Liver Index

As expected, the distribution of the FLI differed between men and women, with fewer women having a high FLI. The elements included in the FLI: triglycerides, BMI, GGT and waist circumference were clearly related with the FLI in both men and women (Table 1). Incident diabetes increased across the FLI groups, *P *< 0.0001 for both men and women, and despite the small numbers of incident cases, the three-yearly incidences also increased across FLI groups. The relation between FLI and diabetes in the family was not statistically significant, but with few exceptions, cardio-metabolic risk factors and liver enzymes were strongly associated with the FLI. Lack of intense physical activity and smoking were also related with the FLI, but for alcohol intake, a positive relation was only clear in the men. The presence of the IDF metabolic syndrome and the mean value of the NAFLD-FLS increased with increasing FLI. The Spearman correlation coefficients between the FLI and the NAFLD FLS were 0.70 in men and 0.64 in women.

**Table 1:** Means ± SD and n (%) of clinical and biological variables associated with incident diabetes, according to values of the Fatty Liver Index of < 20, 20-69, ≥ 70 at baseline; The D.E.S.I.R. Study.

	Men				Women			
	
	Fatty Liver Index			*P*-*values^a^*	Fatty Liver Index			*P*-*values^a^*
	< 20	20-69	≥ 70		< 20	20-69	≥ 70	
	n = 594 (32%)	n = 990 (53%)	n = 277 (15%)		n = 1430 (73%)	n = 445 (23%)	n = 75 (4%)	
Incident diabetes years 0 to 9	14 (2)	69 (7)	57 (21)	0.0001	13 (1)	30 (7)	20 (27)	0.0001
years 0 to 3	5 (1)	21 (2)	26 (10)	0.0001	7 (1)	14 (3)	9 (13)	0.0001
years 3 to 6^b^	3 (1)	18 (2)	16 (7)	0.0001	1 (0.1)	4 (1)	6 (10)	0.0001
years6 to 9^c^	6 (1)	25 (3)	11 (5)	0.005	5 (0.3)	10 (2)	5 (8)	0.0001
Diabetes in family	102 (17)	178 (18)	59 (21)	0.07	274 (19)	104 (23)	17 (23)	0.1
								
Age (years)	43 ± 10	48 ± 10	50 ± 9	0.0001	46 ± 10	52 ± 9	51 ± 9	0.0001
								
Waist circumference (cm)	81 ± 5	91 ± 6	102 ± 7	0.0001	72 ± 6	87 ± 7	101 ± 8	0.0001
BMI (kg/m^2^)	22.5 ± 1.8	25.7 ± 2.0	29.6 ± 2.9	0.0001	22.2 ± 2.3	27.7 ± 2.9	33.4 ± 4.2	0.0001
								
Glucose (mmol/l)	5.3 ± 0.5	5.5 ± 0.5	5.7 ± 0.6	0.0001	5.0 ± 0.4	5.3 ± 0.5	5.6 ± 0.5	0.0001
HbA1c (%)	5.4 ± 0.4	5.5 ± 0.4	5.6 ± 0.4	0.0001	5.3 ± 0.4	5.5 ± 0.4	5.7 ± 0.5	0.0001
Insulin (pmol/l) ‡	33 ± 14	49 ± 24	74 ± 44	0.0001	37 ± 16	58 ± 29	90 ± 44	0.0001
								
Menopause					453 (32)	251 (57)	42 (56)	0.7
Large baby > 4 kg					190 (14)	91 (21)	19 (27)	0.0001
								
Treatment for HTA	15 (3)	102 (10)	51 (18)	0.0001	105 (7)	95 (21)	25 (33)	0.0001
Systolic BP (mmHg)	128 ± 12	135 ± 14	142 ± 16	0.0001	125 ± 14	135 ± 16	141 ± 18	0.0001
Diastolic BP (mmHg)	78 ± 8	82 ± 9	87 ± 10	0.0001	76 ± 8	82 ± 9	83 ± 8	0.0001
Heart rate (min)	64 ± 10	66 ± 9	70 ± 11	0.0001	68 ± 9	69 ± 10	72 ± 12	0.0001
								
Treatment for lipids	22 (4)	90 (9)	33 (12)	0.005	75 (5)	53 (12)	10 (13)	0.2
Triglycerides (mmol/l)^d^	0.79 ± 0.30	1.36 ± 0.74	2.20 ± 1.25	0.0001	0.79 ± 0.35	1.30 ± 0.57	1.88 ± 0.81	0.0001
HDL-cholesterol (mmol/l) ^d^	1.64 ± 0.41	1.44 ± 0.35	1.33 ± 0.31	0.0001	1.86 ± 0.42	1.63 ± 0.39	1.47 ± 0.30	0.0001
Cholesterol (mmol/l)	5.43 ± 0.85	5.92 ± 0.93	6.39 ± 1.07	0.0001	5.47 ± 0.91	6.01 ± 0.99	6.26 ± 1.02	0.0001
								
GGT (IU/l) ^d^	22.2 ± 11.4	40.4 ± 28.1	85.9 ± 76.4	0.0001	17.6 ± 10.9	30.8 ± 23.8	58.8 ± 68.3	0.0001
ALT (IU/l) ^d^	22.8 ± 8.5	32.2 ± 19.3	45.4 ± 25.4	0.0001	17.9 ± 8.3	26.4 ± 22.9	31.4 ± 20.7	0.0001
AST (IU/l) ^d^	20.4 ± 6.3	23.2 ± 11.0	27.8 ± 15.5	0.0001	17.3 ± 6.8	20.4 ± 13.6	21.4 ± 11.2	0.0001
								
Creatinine (μmol/l)	87.2 ± 10.4	89.9 ± 11.1	91.1 ± 13.2	0.0001	73.7 ± 9.6	75.2 ± 10.8	78.8 ± 12.3	0.0001
White Blood Cell count (10^4^/mm^3^) ^d^	62 ± 17	65 ± 17	66 ± 17	0.0001	61 ± 16	65 ± 15	74 ± 36	0.0001
								
Intense physical activity	180 (30)	193 (19)	40 (14)	0.0001	318 (22)	64 (14)	12 (16)	0.01
Smoker	155 (26)	240 (24)	75 (27)	0.03	200 (14)	47 (11)	12 (16)	0.03
Alcohol (g/day)	18 ± 20	25 ± 22	36 ± 29	0.0001	7 ± 11	8 ± 13	9 ± 14	0.8
≥ 30/20g/day men/women	143 (24)	315 (32)	133 (48)	0.0001	363 (25)	125 (28)	23 (31)	0.7
								
IDF metabolic syndrome	0 (0)	162 (17)	193 (71)	0.0001	23(2)	152 (34)	52 (69)	0.0001
NAFLD Fatty Liver Score (5)	-2.34 ± 0.44	-1.57 ± 0.89	-0.14 ± 1.31	0.0001	-2.44 ± 0.57	-1.34 ± 1.06	-0.13 ± 1.21	0.0001

^a ^*p-values *from ANOVA or logistic regression, adjusted on age

^b ^4 individuals with unknown diabetic status at 3 years of follow-up

^c ^7 individuals with unknown diabetic status known at 6 years of follow-up

^d ^log-transformation for analysis because of a non-symmetric distribution

### Relation between incident diabetes and the Fatty Liver Index

The unadjusted odds ratios (95% confidence intervals) for incident diabetes were in men: 10.73 (5.86-19.66) and 3.10 (1.73-5.56) for FLI ≥ 70 and between 20 and 69, respectively, in comparison to men with a FLI < 20; for women, the corresponding odds ratios were 39.64 (18.75-83.78) and 7.88 (4.07 15.24) (Table 2). These odds ratios were slightly attenuated after adjustment for age, and then additionally after separate adjustment for, glucose, insulin, physical activity, smoking, alcohol intake, diabetes in the family, and hypertension. After multivariate adjustment for all of these factors, there was a trend across the three FLI categories. In comparison to those with an FLI < 20, men with a FLI ≥ 70 had an odds ratio for incident diabetes of 3.43 (1.61-7.28), and women 11.05 (4.09-29.81). For the middle group with an FLI of 20-69, the odds ratios differed from 1, and were of the order of two in men and three in women.

**Table 2:** Odds ratios (95% confidence intervals) for incident diabetes, with *P *values for trend across baseline Fatty Liver Index classes; The D.E.S.I.R. Study.

MEN				
	**Fatty Liver Index**			***P *- value ** **trend**
		
**Adjustment factors**	**< 20**	**20-69**	**≥ 70**	
	**n = 594**	**n = 990**	**n = 277**	

Not adjusted	1	3.10 (1.73-5.56)	10.73 (5.86-19.65)	0.0001
Age	1	2.77 (1.54-5.01)	9.33 (5.05-17.25)	0.0001
Age + glucose	1	2.39 (1.28-4.45)	6.09 (3.17-11.70)	0.0001
Age + insulin ‡	1	1.85 (1.00-3.42)	4.28 (2.15-8.50)	0.0001
Age + physical activity	1	2.76 (1.53-5.00)	9.27 (5.00-17.19)	0.0001
Age + smoking	1	2.73 (1.51-4.93)	9.02 (4.87-16.71)	0.0001
Age + alcohol	1	2.65 (1.47-4.80)	8.15 (4.36-15.23)	0.0001
Age + diabetes in family	1	2.77 (1.53-5.00)	9.27 (5.01-17.15)	0.0001
Age + hypertension (HTA)	1	2.58 (1.42-4.68)	7.96 (4.25-14.93)	0.0001
Age + alcohol, glucose, insulin, physical activity, smoking, diabetes in family, HTA	1	1.92 (1.00-3.66)	3.43 (1.61-7.28)	0.0009
				
**Where alcohol < 30 g/day**	n = 451	n = 675	n = 144	

Age	1	3.65 (1.69-7.86)	9.49 (4.12-21.85)	0.0001
Age + alcohol, glucose, insulin, physical activity, smoking, diabetes in family, HTA	1	2.72 (1.16-6.38)	4.71 (1.68-13.16)	0.003

**WOMEN**				

	**Fatty Liver Index**			***P *-value trend**
		
**Adjustment factors**	**< 20**	**20-69**	**≥ 70**	
	**n = 1430**	**n = 445**	**n = 75**	

Not adjusted	1	7.88 (4.07-15.24)	39.64 (18.75-83.78)	0.0001
Age	1	7.21 (3.64-14.28)	36.72 (17.12-78.76)	0.0001
Age + glucose	1	3.79 (1.84-7.77)	13.60 (5.92-31.24)	0.0001
Age + insulin ‡	1	4.65 (2.22-9.72)	15.49 (6.17-38.85)	0.0001
Age + physical activity	1	7.08 (3.57-14.06)	36.06 (16.78-77.48)	0.0001
Age + smoking	1	7.12 (3.59-14.11)	35.70 (16.62-76.68)	0.0001
Age + alcohol	1	7.23 (3.65-14.33)	37.13 (17.28-79.78)	0.0001
Age + diabetes in family	1	6.87 (3.45-13.70)	37.87 (17.45-82.17)	0.0001
Age + hypertension (HTA)	1	6.42 (3.21-12.85)	29.24 (13.22-64.68)	0.0001
Age + alcohol, glucose, insulin, physical activity, smoking, diabetes in family, HTA	1	3.27 (1.49-7.20)	11.05 (4.09-29.81)	0.0001
				
**Where alcohol < 20 g/day**	n = 1067	n = 320	n = 52	

Age	1	9.79 (4.02-23.85)	66.84 (25.70-173.88)	0.0001
Age + alcohol, glucose, insulin, physical activity, smoking, diabetes in family, HTA	1	4.38 (1.62-11.83)	22.77 (6.78-76.44)	0.0001

### Relations in the population without an excessive alcohol intake

When the analyses were restricted to men with an alcohol intake < 30 g/day and women with an intake < 20 g/day, the fully adjusted odds ratios for incident diabetes in those with an FLI ≥ 70 were increased to 4.71 (1.68-13.16) in men and 22.77 (6.78-76.44) in women, in comparison to men or women with a FLI < 20. (Table 2).

### Comparing the predictive power of the Fatty Liver Index and the NAFLD Fatty Liver Score

To compare these two fatty liver incides, the population was divided into sex-specific quartiles for each index. The unadjusted odds ratios showed that both the FLI and the NAFLD-FLS were highly predictive of incident diabetes (Table 3). In comparison to the first quartile group, the last quartile group of the FLI carried odds ratios of 11.69 (5.58-6.74) in men and 54.40 (7.49-395) in women; for the NAFLD-FLS the odds were 9.47 (4.84-18.52) and 27.13 (6.56-112) respectively. After adjustment on diabetes risk factors, the odds ratios for incident diabetes increased across the quartiles, with the odds ratios for last quartile groups higher than the odds ratios for the first quartile groups, which were chosen as the reference groups. Comparing the highest quartile group of these indices with the group including individuals in the three lower quartile groups, after adjustment for other factors associated with incident diabetes, the two indices provided similar odds ratios - for men of the order of 2, and for women, 4. However, the odds ratios did not differ statistically between men and women, (FLI: *P *= 0.2 and NAFLD-FLS: *P *= 0.1).

**Table 3:** Odds ratios (95% confidence intervals) for incident diabetes, across sex specific quartile groups of Fatty Liver Index and NAFLD fatty liver at baseline; The D.E.S.I.R. Study.

MEN		no adjustment		with adjustment*	
		
	diabetics/n	OR (95% CI)	*P*	OR (95% CI)	*P*
**Fatty Liver Index**					
< 15.52	8/466	1		1	
15.52 - 33.38	23/464	**2.99 [1.32 - 6.74]**	0.008	1.92 [0.81 - 4.56]	0.1
33.39 - 57.67	30/465	**3.95 [1.79 - 8.71]**	0.0007	2.17 [0.92 - 5.11]	0.07
> 57.67	79/466	**11.69 [5.58 - 24.48]**	0.0001	**3.89 [1.65 - 9.14]**	0.002
**Fatty Liver Index**					
≤ 57.67	61/1395	1		1	
**>**57.67	79/466	**4.46 [3.14 - 6.35]**	0.0001	**2.04 [1.30 - 3.20]**	0.002
**NAFLD fatty liver score**					
< -2.34	10/462	1		1	
-2.34 - -1.94	22/462	**2.26 [1.06 - 4.83]**	0.03	1.97 [0.88 - 4.37 ]	0.1
-1.93 - -1.15	25/462	**2.59 [1.23 - 5.45]**	0.01	2.05 [0.94 - 4.51 ]	0.07
> -1.15	80/462	**9.47 [4.84 - 18.52]**	0.0001	**3.98 [1.93 - 8.20]**	0.0002
**NAFLD fatty liver score**					
≤ -1.15	57/1386	1		1	
> -1.15	80/462	**4.88 [3.41 - 6.99]**	0.0001	**2.32 [1.53 - 3.50]**	0.0001

**WOMEN**		**no adjustment**		**with adjustment**^a^	
		
	**diabetics/n**	**OR (95% CI)**	***P***	**OR (95% CI)**	***P***

**Fatty Liver Index**					
< 3.78	1/488	1		1	
3.78 - 7.64	4/487	4.03 [0.45 - 36.12]	0.2	3.51 [0.38 - 32.13]	0.3
7.65 - 21.64	9/488	**9.14 [1.15 - 72.30]**	0.04	4.09 [0.49 - 33.92]	0.2
> 21.64	49/487	**54.40 [7.49 - 395]**	0.0001	**12.78 [1.59 - 102]**	0.02
**Fatty Liver Index**					
≤ 21.64	14/1463	1		1	
> 21.64	49/487	**11.58 [6.33 - 21.17]**	0.0001	**3.78 [1.79 - 7.95]**	0.0005
NAFLD fatty liver score					
< -2.65	2/486	1		1	
-2.65 - -2.33	7/487	3.53 [0.73 - 17.07]	0.1	2.81 [0.57 - 13.92 ]	0.2
-2.32 - -1.82	4/487	2.00 [0.37 - 10.99]	0.4	1.17 [0.20 - 6.71 ]	0.9
> - 1.82	49/486	**27.13 [6.56 - 112]**	0.0001	**6.58 [1.48 - 29.26]**	0.01
**NAFLD fatty liver score**					
≤ -1.82	13/1460	1		1	
> - 1.82	49/486	**12.48 [6.71 - 23.22]**	0.0001	**3.95 [1.93 - 8.07]**	0.0002

^a ^adjusted for age, glucose, insulin, physical activity, smoker, alcohol, diabetes in family, HTA; the NAFLD-FLS is not adjusted for insulin as insulin is already included in the NAFLD-FLS index

The fully adjusted FLI provided a reasonable discrimination of cases and non-cases of incident diabetes, with c-statistics of 0.74 in men and 0.84 in women and for the NAFLD-FLS the c-statistics were 0.74 and 0.83, respectively; the models provided a reasonable fit according to the Hosmer-Lemeshow test, with all tests non-significant.

As expected, the models we have already published, which included the best combination of clinical and biological factors to predict diabetes, had higher c-statistic values, 0.85 in men and 0.92 in women. The fatty liver indices and the D.E.S.I.R. diabetes score screen different high risk individuals: those with a high score from the D.E.S.I.R. risk score have higher glucose and HbA1c levels but have less adiposity, lower liver function tests and lower insulin levels than those at high risk according to the FLI and the NAFLD-FLS indices.

## Discussion

These two fatty liver indices were both predictive of incident diabetes, after adjusting for other known risk factors of diabetes, including the most predictive factor, fasting glucose at baseline. For the 15% of men with a FLI > 70, the multivariate adjusted odds ratio for incident diabetes was over three and for the 4% of women, the odds ratio was over ten, however, this difference was not statistically significant. In people with moderate alcohol intake, the odds ratios were higher. While the individual factors in the FLI - GGT, triglycerides, waist circumference and BMI - have all been shown to be related with incident diabetes in our cohort [16], this specific linear combination of diabetes risk factors, reflecting fatty liver, is also related with incident diabetes. Equally, the NAFLD-FLS also predicted incident diabetes in both sexes. Again, most of the individual parameters in this score are predictors of incident diabetes in univariate analysis in our cohort [16], the exception being AST/ALT which we have not studied.

In the Korean study of 5372 individuals [12], of whom 68% were men, the relative risk of 5-year incident diabetes in ultrasound diagnosed fatty liver, in comparison with those without fatty liver, was 2.3 (1.1 4.7) in the 9% of the population with moderate to severe fatty liver, and 1.5 (0.8-2.8) in the 21% with mild fatty liver, after adjustment for other diabetes risk factors, including baseline fasting glucose. It is not clear whether the authors adjusted for alcohol intake, as it is not indicated in the tables, only in the text in the Discussion section. In this report, the authors did not analyse the predictive value of fatty liver separately in men and women. A Chinese case-control study [13] also showed that people with ultrasound-diagnosed NAFLD had a diabetes incidence of 20% in comparison to 5% in those without NAFLD; more than 90% of participants in this study were men. Other studies with smaller series of NAFLD patients have also shown that a fatty liver is predictive of later diabetes [17,18], however these studies must be interpreted with caution, as there was no control group. In contrast, in a large population of American veterans, of whom 98% were men, diabetes at baseline was related with NAFLD ten years later [19]. As the prevalent cases of NAFLD were included in the analysis, the time sequence of NAFLD and diabetes cannot be determined from this study [19].

One of the proposed mechanisms for the relation between fatty liver and insulin resistance is hepatic 'fat-overflow', which leads to lipid accumulation and then to hepatic insulin resistance followed by overall insulin resistance [7]. It has been shown in a cross sectional study in the Pima Indian population, that GGT and ALT activities were inversely correlated with both peripheral and hepatic insulin sensitivity, as evaluated by a hyperinsulinaemic, euglycaemic clamp [20]. Another hypothesis is that insulin resistance is invoked by an adipo-cytokine imbalance, which then leads on to NAFLD [7]. Diabetes and insulin resistance could develop at the same time as NAFLD. Comparing the logistic regression models from our study, adjusted on age and then age and insulin, there was a reduction in the odds ratios for incident diabetes associated with a high FLI ≥ 70 from 9.33 to 4.28 in men, and from 36.72 to 15.49 in women, still significantly higher than in the group with FLI < 20. Thus the relation was attenuated, but not fully accounted for, by high insulin levels. A higher FLI may reflect enhanced hepatic gluconeogenesis, which is known to contribute to the pathophysiology of dysglycemia. The FLI may be viewed as an indirect marker of impaired insulin secretion, one of the major determinants of incident diabetes [21]. Early interactions between beta cells and the liver need further study.

We must acknowledge the limitation of using indices of fatty liver in our study, as we did not have access to scanning devices. In large epidemiologic studies, such techniques are rarely available. The FLI has been developed using ultrasound, the NAFLD-FLS by proton magnetic resonance spectroscopy, whereas biopsy is the gold standard. Both scores can be criticised, and in particular the NAFLD-FLS includes a large number of correlated items as the metabolic syndrome is included, as well as diabetes and insulin, and also both AST and AST/ALT. Further, the populations on which these two studied indices were developed are particular populations: for the FLI, 50% of the individuals had suspected liver disease, and for the NAFLD-FLS, one quarter of the individuals had diabetes. These selection criteria led to average BMIs of 27 kg/m^2 ^and over 30 kg/m^2^, for the populations in which the FLI and the NAFLD-FLS were developed; the average BMI is close to 25 kg/m^2 ^in our French study. We are not able to evaluate the sensitivity, specificity and predictive values of either of these fatty liver indices in our general French population, as we do not have a precise evaluation of fatty liver. The strengths of our study include the large size of the study population, the fact that it is a general population not selected for particular characteristics, the long nine year follow-up and the concordant results in both men and women.

## Conclusions

The novel finding of our study is that these two recently published indices of liver fat are predictive of diabetes in both men and women. The association was independent of traditional risk factors for diabetes such as glucose, diabetes in the family, insulin, sedentarity, smoking and alcohol consumption, suggesting the clinical interest of these indices to better identify patients consulting hepatologists, who are at high risk of progression to diabetes.

## Abbreviations

ALT: alanine aminotransferase; AST: asparate aminotransferase; BMI: Body Mass Index; D.E.S.I.R.: Data from an Epidemiological Study on the Insulin Resistance syndrome; FLI: Fatty Liver Index; GGT: gamma glutamyl transferase; HTA: hypertension; IDF: International Diabetes Federation; NAFLD-FLS: non alcoholic fatty liver disease - fatty liver score; NASH: non alcoholic steatohepatitis.

## Competing interests

The authors declare that they have no competing interests.

## Authors' contributions

BB is the principal investigator of the D.E.S.I.R. study, designed the statistical analysis for this study, and wrote the article; CL did the statistical analysis and critically read the manuscript; SV was involved in the conception of the D.E.S.I.R. study and critical reading of the manuscript; FF was involved in the conception of the D.E.S.I.R. study and critical reading of the manuscript; FB assisted in the design of the statistical analysis, the writing and critical reading of the manuscript. All authors read and approved the final manuscript.

## Pre-publication history

The pre-publication history for this paper can be accessed here:

http://www.biomedcentral.com/1471-230X/10/56/prepub
